# Lifestyle Factors Associated with Circulating Very Long-Chain Saturated Fatty Acids in Humans: A Systematic Review of Observational Studies

**DOI:** 10.1016/j.advnut.2022.10.004

**Published:** 2022-12-17

**Authors:** Kira Zhi Hua Lai, Nagam A. Yehia, Zhila Semnani-Azad, Sonia Blanco Mejia, Beatrice A. Boucher, Vasanti Malik, Richard P. Bazinet, Anthony J. Hanley

**Affiliations:** 1 Department of Nutritional Sciences, Faculty of Medicine, University of Toronto, Toronto, Ontario, Canada; 2 Department of Nutrition, Harvard T.H. Chan School of Public Health, Boston, MA, USA; 3 Toronto 3D Knowledge Synthesis and Clinical Trials Unit, Risk Factor Modification Centre, St Michael’s Hospital, Toronto, Ontario, Canada; 4 Division of Endocrinology and Metabolism, University of Toronto, Toronto, Ontario, Canada; 5 Leadership Sinai Centre for Diabetes, Mount Sinai Hospital, Toronto, Ontario, Canada

**Keywords:** very long-chain saturated fatty acids, arachidic acid, behenic acid, lignoceric acid, diet, smoking, physical activity, alcohol consumption

## Abstract

Recent observational studies have documented inverse associations of circulating very long-chain saturated fatty acids (VLCSFAs), namely arachidic acid (20:0), behenic acid (22:0), and lignoceric acid (24:0), with cardiometabolic outcomes. In addition to their endogenous production, it has been suggested that dietary intake or an overall healthier lifestyle may influence VLCSFA concentrations; however, a systematic review of the modifiable lifestyle contributors to circulating VLCSFAs is lacking. Therefore, this review aimed to systematically assess the effects of diet, physical activity, and smoking on circulating VLCSFAs. Following registration on PROSPERO (International Prospective Register of Systematic Reviews) (ID: CRD42021233550), a systematic search of observational studies was conducted in MEDLINE, EMBASE, and The Cochrane databases up to February 2022. A total of 12 studies consisting of mostly cross-sectional analyses were included in this review. The majority of the studies documented the associations of dietary intake with total plasma or red blood cell VLCSFAs, in which a range of macronutrients and food groups were examined. Two cross-sectional analyses showed a consistent positive association between total fat and peanut intake with 22:0 and 24:0 and an inverse association between alcohol intake and 20:0 and 22:0. Furthermore, a moderate positive association between physical activity and 22:0 and 24:0 was observed. Lastly, there were conflicting results on the effects of smoking on VLCSFA. Although most studies had a low risk of bias; the findings of this review are limited by the bi-variate analyses presented in the majority of the included studies, therefore, the impact of confounding is unclear. In conclusion, although the current observational literature examining lifestyle determinants of VLCSFAs is limited, existing evidence suggests that circulating 22:0 and 24:0 may be influenced by higher total and saturated fat consumption and nut intake.

**Statement of significance**

This systematic review summarizes current evidence on the lifestyle and dietary variables that influence circulating levels of arachidic (20:0), behenic (22:0), and lignoceric (24:0) acids in humans. In addition, the review identified significant gaps in the literature, which may prompt future studies on these unique, very long-chain saturated fatty acids.

## Introduction

Circulating saturated fatty acids are involved in multiple complex metabolic and biological functions and have been shown to be markers of dietary intake and metabolic health [1]. Recent studies have highlighted that saturated fatty acids of different carbon chain lengths have varying metabolic effects and are associated with a range of health outcomes [[2], [3], [4], [5]]. Specifically, meta-analyses of prospective cohort studies have shown that a unique group of saturated fats, namely arachidic acid (20:0), behenic acid (22:0), and lignoceric acid (24:0), are associated with a lower incidence of type 2 diabetes and cardiovascular diseases [6, 7]. Additionally, well-characterized prospective cohort studies have shown that 20:0, 22:0, and 24:0 are associated with an improved healthy lifespan [8], and 22:0 and 24:0 are associated with a lower risk of all-cause mortality [9].

The fatty acids 20:0, 22:0, and 24:0 are categorized as very long-chain saturated fatty acids (VLCSFAs), which are commonly defined as saturated fatty acids with a chain length of ≥20 carbons [10]. Other longer-chain VLCSFAs (26–38 carbons) are present within the skin and the ocular surface, the meibum [11], but are not widely found within the bloodstream of healthy individuals; hence, they will not be considered in this study. VLCSFAs are mainly produced endogenously through the elongation of long-chain saturated fatty acids within the endoplasmic reticulum [12]. It has been suggested that circulating VLCSFAs may not be strongly influenced by dietary sources, given the diminished absorption rate of fatty acids with increasing chain length [13]. However, intervention studies using foods high in VLCSFAs, such as peanuts and macadamia nuts, have shown that long-term or acute intake of these foods increases the plasma concentration of VLCSFAs [[14], [15], [16]]. Furthermore, observational studies have suggested that healthier lifestyle habits, including higher physical activity, lower alcohol consumption, and smoking rates, are associated with higher circulating concentrations of VLCSFAs [[17], [18], [19]].

In light of the potential health benefits of VLCSFAs, there is increasing interest in understanding the modifiable factors associated with their circulating concentrations. However, to date, the literature on this topic has not been systematically reviewed. Therefore, this systematic review aimed to summarize the lifestyle predictors of circulating VLCSFAs in humans, specifically focusing on 20:0, 22:0, and 24:0.

## Methods

This review was conducted according to the PRISMA (Preferred Reporting Items for Systematic Reviews and Meta-Analyses) guidelines [20] and was registered on the PROSPERO (Prospective Register of Systematic Reviews) prior to the inception of the review (ID: CRD42021233550).

### Search strategy

We searched MEDLINE, EMBASE, and The Cochrane Central Register of Controlled Trials from the inception of the databases to 4th February 2022 (Supplementary Table 1). We searched for relevant primary observational studies that assessed the association of lifestyle variables (diet, physical activity, and smoking status) with circulating VLCSFAs of interest, namely 20:0, 22:0, and 24:0. Manual searches supplemented the electronic database searches, including reference lists of selected studies and review articles.

### Study selection and data extraction

The inclusion criteria for this systematic review included: *1*) observational studies; *2*) full-text available; *3*) reporting on associations of lifestyle variables with circulating VLCSFA concentrations; *4*) original articles in humans. Exclusion criteria included: *1*) case reports or reviews; *2*) experimental studies, *3*) animal studies; *4*) in vitro or in vivo studies; *5*) studies that exclusively focused on subjects with chronic diseases or genetic disorders that may affect lipid metabolism.

The prespecified primary outcome of interest was the concentration or proportions of 20:0, 22:0, or 24:0 in blood, serum, or plasma. One reviewer (KZHL) extracted relevant data using a standardized form. Extracted data captured the following information: first author’s last name, year of publication, original study design, population characteristics (sample size, sex, age, and study duration), fatty acid species documented, the serum/plasma lipid fraction, fatty acids unit of measure (% total fatty acid and concentration), lipid quantification methods, an instrument used for diet assessment, variables evaluated for association with VLCSFAs, relevant results, and statistical methods and covariates adjusted in multivariable analyses. The extracted data for each study was evaluated by a second reviewer (AJH).

### Risk of bias assessments

Two independent reviewers (KZHL and NAY) assessed the included studies for the risk of bias. The Newcastle-Ottawa-Scale (NOS) was used to evaluate the quality of case-control and prospective cohort studies [21], and a modified version of NOS by Herzog et al. [22] was used for cross-sectional studies. Both scoring systems examined the quality of studies based on the selection of the cohort population, the comparability of the studied groups, and the ascertainment of either the exposure or outcome of interest for case-control or cohort studies. A maximum of 10 points was given to cross-sectional studies and 9 points for prospective cohort and case-control studies, and a score of 6 and above was considered a low risk of bias. All disagreements were reconciled by consensus.

## Results

### Identification of studies

**FIGURE 1:**
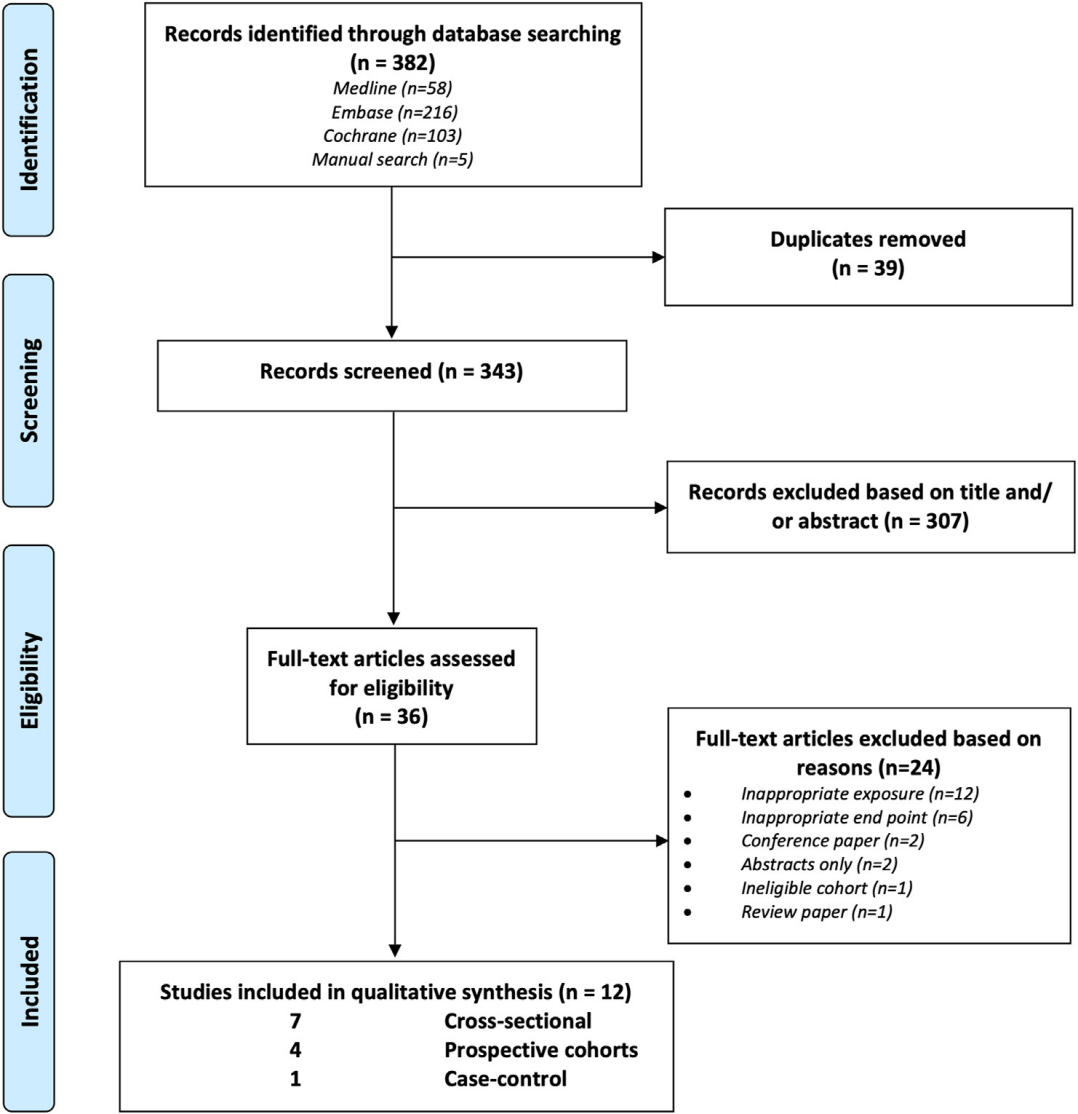
PRISMA (Preferred Reporting Items for Systematic Reviews and Meta-Analyses) flow diagram of included studies examining the lifestyle correlates of circulating very long-chain fatty acids (VLCSFAs).

Figure 1 illustrates the flow diagram of the search strategy and study selection. Of the 382 articles identified through the search, 39 articles were duplicates, and 307 articles did not meet inclusion criteria after screening titles and abstracts. The full texts of the remaining 36 articles were reviewed, of which 24 articles did not meet the inclusion criteria. Thus, a total of 12 observational studies were included.

### Study characteristics

Of the included articles, there were 7 cross-sectional studies [[23], [24], [25], [26], [27], [28], [29]], 4 prospective cohort studies [[17], [18], [19], 30], and 1 case-control study [31]. However, given that lipid metabolism may be affected by medications or the presence of disease or pregnancy status, only results from presumably healthy or nonpregnant controls were extracted from the case-control study [31]. Similarly, only cross-sectional results from baseline disease-free subjects were extracted from 3 of the included prospective cohort studies [[17], [18], [19]], with only 1 longitudinal analysis meeting the inclusion criteria [30]. Hence, the results of this review were extracted from 1 longitudinal analysis [30] and 11 cross-sectional analyses [[17], [18], [19], [23], [24], [25], [26], [27], [28], [29], 31].

The reported results were mainly from healthy participants; however, 7 studies included some participants with a clinical history of metabolic risk phenotypes such as hyperlipidemia, metabolic syndrome, or obesity [[17], [18], [19], 23, 26, 28, 30]. The majority of the studies were conducted in Europe [23, [26], [27], [28], [29], [30]], followed by North America [17, 19, 31], South America [24], Asia [18], and Australia [25]. The sample size of the studies ranged between 139 [25] to 3179 [19] adults.

Most of the fatty acids measured were extracted from the total plasma pool [17, 18, 24, 26, 27, 29], followed by the red blood cell [17, 23, 28, 31], and the phospholipid pool [19, 25, 30]. Lastly, 1 study included serum fatty acids from the cholesteryl ester, triacylglycerol, and nonesterified fatty acid pool [24].

### Dietary intake

Table 1 outlines the studies examining dietary determinants of circulating VLCSFA, and Table 2 summarizes the association of each dietary component with VLCSFAs. Nine studies examined the association of diet with circulating VLCSFAs, of which 8 analyses were cross-sectional [[17], [18], [19], [23], [24], [25], 27, 31], with only 1 longitudinal analysis examining the impact of changes in dietary intake on VLCSFA over 13 years [30]. Most of the studies used a validated FFQ to assess dietary intake [17, 19, [23], [24], [25], 30]; however, 3 studies used other methods, including a validated 12-item fat questionnaire [31], 4-day food diaries [27] or an in-person interview to assess alcohol consumption [18].

**Table 1:** Summary of 9 studies that examined the associations of dietary intake with circulating very long-chain fatty acids (VLCSFA)^1^

Reference	Original study design	*n*, Age	Country	Fatty acid	Serum fraction (methods)	Fatty acid unit	Instruments for diet assessment	Exposure	Results	Statistical analyses/covariates
Ardisson Korat et al., 2020 [17]	Prospective cohort study (Only cross-sectional analyses reported)	2854 (F:1392, M:1462), Mean age: 60-65y	USA	20:0 22:0 24:0	Plasma and RBC (GC-FID)	%	Validated FFQ	Peanut butter, vegetable fat, peanuts, total fat, polyunsaturated fat, coffee, 16:0-, 18:0-diet, dairy fat, other nuts, carbohydrate, animal fat, fruits, vegetables, unprocessed meat, potato chips, alcohol	Baseline cross-sectional analysis from healthy participants: • Intakes of peanuts (*r* = 0.04-0.15)^5^, peanut butter (*r* = 0.10-0.15)^5^ were positively correlated with 22:0 and 24:0 • total fat (*r =* 0.04-0.16)^5^, polyunsaturated fat (*r* = 0.04-0.14)^5^, animal fat (*r* = 0.01-0.10)^5^, vegetable fat (*r* = 0.04-0.15)^5^*,* dairy fat (*r* = 0.05-0.14)^5^, and palmitic & stearic acids (*r* = 0.04-0.16)^5^ were positively correlated with all VLCSFA • There are conflicting associations between vegetable and fruit intake with VLCSFA • Alcohol intake was inversely correlated with 20:0 and 22:0 (*r* = -0.05 to -0.10)^5^ • Coffee intake was positively correlated with all VLCSFA (*r* = 0.05 – 0.10)^5^	Spearman correlation/adjusted for age, total energy intake, BMI
Furtado et al., 2019 [24]	Cross-sectional	200 (F: 101, M:99), Mean age: 57-62y	Costa Rica	20:0 22:0 24:0	Total plasma, PL, CE, TAG+NEFA (GC-FID)	%	Validated semi-quantitative FFQ modified for the Costa Rican population	Dietary 20:0, Dietary 22:0	• No significant correlations between dietary 20:0 and 22:0 with plasma 20:0 and 22:0 (intake of 20:0 and plasma 20:0 (*r* = 0.08), intake of 22:0 and plasma 22:0 (*r* = 0.04)	Spearman correlation adjusted for age, sex, BMI, dietary supplement use
Lemaitre et al., 2014 [31]	Case-control (only reported results from healthy controls)	Healthy controls: 415 (M:81%), Mean age 58y	USA	20:0 22:0 24:0	RBC (GC-FID)	%	Validated 12-item fat questionnaire (Northwest lipid research clinical fat intake scale)[32]	Saturated fat intake	Results in controls: • 22:0 and 24:0 were positively associated with index of fat intake (*r* = 0.20)^5^	Correlation
Lemaitre et al., 2015 [19]	Prospective cohort study (Only cross-sectional analyses reported)	3179 (M:38.6%), Mean age: 75y	USA	20:0 22:0 24:0	PL (GC-FID)	%	Validated FFQ	Total fat, polyunsaturated fat, saturated fat, carbohydrate, protein intake, peanut intake (serving/week) (All food intake data are presented as % energy), alcohol	Baseline cross-sectional analysis from healthy participants: • Total fat, PUFA and peanut intake were positively associated with 22:0 and 24:0 [fat (Q1 of 22:0: 31% energy vs Q4:33%), (Q1 of 24:0 31% vs 33%), PUFA (Q1 of 22:0: 7% vs 7.7%),(Q1 of 24:0: 7.1% vs Q4:7.7%), peanut(Q1 of 22:0 1.1 serving/week vs 1.8), (Q1 of 24:0: 1.0 vs 1.9)]^3^ • Saturated fat is positively associated with 20:0 (Q1 of 20:0: 9.9% energy vs 10.3% )^3^ and 22:0 (Q1 of 22:0: 9.8% vs 10.4%)^3^ • Total protein intake was inversely associated with 22:0 (Q1 of 22:0: 19.2% energy vs 18.7%)^3^ • Carbohydrate is negatively associated with all VLCSFA (Q1 of 20:0: 53.5% energy vs 52.1%), (Q1 of 22:0: 53.8 vs 52.0%),(Q1 of 24:0: 53.7% vs 52.2%)^3^ • Alcohol consumption was inversely associated with 20:0 and 22:0 (Q1 of 20:0: 6.1 vs Q4: 3.1), (Q1 of 22:0:6.2 vs Q4:3.0)^3^	Linear regression
Li., 2001 [25]	Cross-sectional	139 (M:139), Age range: 20-55 y	Australia	20:0	PL (GC-FID)	Concentration (mg/100 ml)	Validated semi-quantitative FFQ	Meat intake (high meat eaters, moderate meat, ovolacto vegetarians, vegans)	• Ovolacto and vegan diets had a significantly lower 20:0 compared to moderate and high meat eaters [20:0 (ovolacto/vegan: 0.1-0.2mg/100ml vs meat/ moderate meat: 0.3-0.4)]^3^	ANOVA and Fisher’s post hoc
Takkunen et al., 2013 [23]	Cross-sectional	1008, (M:1008), (Mean age 63);	Finland	20:0 22:0 24:0	RBC (GC)	%	Validated FFQ	Saturated fat from milk and milk products, spreads and cooking fat. Polyunsaturated fat from fish and fish oil supplements, spreads and cooking fat, meat products	• Polyunsaturated fat from fish/fish oil supplement intake negatively correlated with 20:0 (*r* =-0.07) 2, and 22:0 (*r* = -0.182)^4^ • Saturated fat from milk products (*r* = 0.132)^4^, spreads and cooking fat (*r* = 0.086)^3^ and polyunsaturated fat from spreads and cooking fat (*r* = -0.089) 3, were correlated with 22:0.	Spearman correlation
Zhao et al., 2017 [27]	Cross-sectional	601 (M:239, F:362), Mean age: 39.1-41.4y	Ireland	20:0 22:0 24:0	Plasma (GC-MS)	%	4-day food diary	Vitamin E intake from food sources	• No significant associations (20:0 (vitamin E intake Q1: 0.20% vs Q4: 0.20%), 22:0 (Q1: 0.33% vs Q4:0.31%), 24:0(Q1: 0.26% vs Q4: 0.24%))	General linear models, adjusted for gender, vitamin E containing supplement use, total energy intake and corresponding fatty acid intake
Zhao et al., 2018 [18]	Cross-sectional	1729 (M; 586, F:1143) (Age:35-59y)	China	20:0 22:0 24:0	Plasma (GC-FID)	%	In-person interview	Alcohol consumption (at least once a week during the past year)	• The highest quartiles of 20:0 and 22:0 had fewer subjects that consumed alcohol [(Q1 20:0: 133 subjects vs Q4: 104)^2^ (Q1 22:0 146 subjects vs Q4:104)^3^]	χ2 test
Zheng et al., 2019 [30]	Longitudinal (Baseline and 2 follow-up visits over 13 years)	722 (M:39.1%), Mean age: 56y	UK	20:0, 22:0, 24:0	PL (GC-FID)	%	Validated semi-quantitative FFQ at each clinic visit	Fruit, vegetable, legume, total dairy, egg, white fish, fatty fish, red meat, white meat, processed meat, liver, potatoes, cereal, bread, sweets, nuts and seeds, tea, coffee, fruit juice, sugar-sweetened beverage, margarine, vegetable oil, alcohol	• Increased servings of fatty fish, white meat, margarine, and vegetable oil resulted in +13.3%^2^, -11.3%^2^, -1.72%^3^, -2.16%^3^ change/year in 20:0 • Increased servings of legumes, potatoes, bread, nuts and seeds, and vegetable oil result in *-*11.8%^3^, +4.26%^3^, -4.86%^3^, +2.85%^2^ and -1.36%^2^ change/year in 22:0 • Increased servings of legumes, white fish, white meat, potatoes, bread, nuts and seeds, and vegetables result in -9.03%^2^, +13.2%^2^, -9.21%^2^,+3.42%^2^, -3.38%^2^, +2.53%^2^, -1.63%^3^ change/year in 24:0	Mixed-effects linear regression models, adjusted for year of recruitment, sex, baseline age, BMI, physical activity, smoking, alcohol drinking, educational level, social class, total energy intake, fish oil supplements, corresponding fatty acid group, and food group, and the change in all the other food groups and their interactions with follow-up duration

1 CE: cholesteryl ester; FFQ: food frequency questionnaire; GC-FID: Gas chromatography – flame ionization detector; GC-MS: Gas chromatography-mass spectrometry; NEFA: non-esterified fatty acids; PL: Phospholipid; RBC: red blood cell; TAG: triacylglycerol; UK: United Kingdom; USA: United States of America.

2 *p* < 0.05.

3 *p* < 0.01.

4 *p* < 0.001.

5 did not specify p-value.

**Table 2:** Summary of the associations between dietary intake with VLCSFA from 9 studies^1^

Determinants		References		
		Positive association	Negative association	No association
**Nutrients**				
Total fat (*n* = 2)	20:0	[17] 2	—	[19] 3
	22:0	[17] 2, [19] 3	—	—
	24:0	[17] 2, [19] 3	—	—
Saturated fat (*n* = 3)	20:0	[19] 3	—	[23] 4^,^^7^, [31] 4
	22:0	[19] 3, [23] 4^,^^7^, [31] 4	—	—
	24:0	[31] 4	—	[23]^4^^,^^7^, [19] 3
Dietary 16:0 or 18:0 (*n* = 1)	20:0	[17] 2	—	—
	22:0	[17] 2	—	—
	24:0	[17] 2	—	—
Dietary 20:0 or 22:0 (*n* = 1)	20:0	—	—	[24] 6
	22:0	—	—	[24] 6
	24:0	—	—	n/a
Polyunsaturated fat (*n* = 3)	20:0	[17] 2	[23] 4^,^^7^	[19] 3
	22:0	[17] 2, [19] 3	[23] 4^,^^7^	—
	24:0	[17] 5, [19] 3	—	[23] 4^,^^7^
Dairy fat (*n* = 1)	20:0	[17] 2	—	—
	22:0	[17] 2	—	—
	24:0	[17] 2	—	—
Animal fat (*n* = 1)	20:0	[17] 2	—	—
	22:0	[17] 2	—	—
	24:0	[17] 5	—	—
Carbohydrate (*n* = 2)	20:0	—	[19] 3	[17] 2
	22:0	—	[19] 3	[17] 2
	24:0	—	[19] 3	[17] 2
Protein (*n* = 1)	20:0	—	—	[19] 3
	22:0	—	[19] 3	
	24:0	—	—	[19] 3
Vitamin E (*n* = 1)	20:0	—	—	[27] 5
	22:0	—	—	[27] 5
	24:0	—	—	[27] 5
**Food and whole foods groups**				
Meat (*n* = 1)	20:0	[25] 3^,^^8^	—	—
	22:0	—	—	—
	24:0	—	—	—
Red meat (*n* = 1)	20:0	—	—	[30] 3
	22:0	—	—	[30] 3
	24:0	—	—	[30] 3
White meat (*n* = 1)	20:0	—	[30] 3	—
	22:0	—	—	[30] 3
	24:0	—	[30] 3	—
Processed meat (*n* = 1)	20:0	—	—	[30] 3
	22:0	—	—	[30] 3
	24:0	—	—	[30] 3
Unprocessed meat (*n* = 1)	20:0	—	—	[17] 2
	22:0	—	—	[17] 2
	24:0	—	—	[17] 2
Fatty fish (*n* = 1)	20:0	[30] 3	—	—
	22:0		—	[30] 3
	24:0	—	—	[30] 3
White fish (*n* = 1)	20:0	—	—	[30] 3
	22:0	—	—	[30] 3
	24:0	[30] 3	—	—
Eggs (*n* = 1)	20:0	—	—	[30] 3
	22:0	—	—	[30] 3
	24:0	—	—	[30] 3
Liver (*n* = 1)	20:0	—	—	[30] 3
	22:0	—	—	[30] 3
	24:0	—	—	[30] 3
Milk products/ dairy (*n* = 1)	20:0	—	—	[30] 3
	22:0	—	—	[30] 3
	24:0	—	—	[30] 3
Potatoes (*n* = 1)	20:0	—	—	[30] 3
	22:0	[30] 3	—	—
	24:0	[30] 3	—	—
Cereal (*n* = 1)	20:0	—	—	[30] 3
	22:0	—	—	[30] 3
	24:0	—	—	[30] 3
Bread (*n* = 1)	20:0	—	—	[30] 3
	22:0	—	[30] 3	—
	24:0	—	[30] 3	—
Fruits (*n* = 2)	20:0	—	—	[17] 2, [30] 3
	22:0	—	—	[17] 2, [30] 3
	24:0	—	—	[17] 2, [30] 3
Vegetables (*n* = 2)	20:0	—	—	[17] 2, [30] 3
	22:0	—	[17] 2	[30] 3
	24:0	—	—	[17] 2, [30] 3
Legumes (*n* = 1)	20:0	—	—	[30] 3
	22:0	—	[30] 3	—
	24:0	—	[30] 3	—
Nuts and seeds (*n* = 2)	20:0	—	—	[30] 3
	22:0	[30] 3	—	—
	24:0	[30] 3	—	—
Peanuts (*n* = 2)	20:0	—	—	[17] 2, [19] 3
	22:0	[17] 5, [19]	—	—
	24:0	[17] 2, [19] 3	—	—
Peanut butter (*n* = 1)	20:0		—	[17] 2
	22:0	[17] 2	—	—
	24:0	[17] 2	—	—
Other nuts (*n* = 1)	20:0	[17] 2	—	—
	22:0	—	—	[17] 2
	24:0	[17] 2	—	—
Margarine (*n* = 1)	20:0	—	[30] 3	—
	22:0	—	—	[30] 3
	24:0	—	—	[30] 3
Vegetable fat/ oil (*n* = 2)	20:0	[17] 2	[30] 3	—
	22:0	[17] 2	[30] 3	—
	24:0	[17] 5	[30] 3	—
Sweets (*n* = 1)	20:0	—	—	[30] 3
	22:0	—	—	[30] 3
	24:0	—	—	[30] 3
Potato chips (*n* = 1)	20:0	—	—	[17] 2
	22:0	[17] 2	—	—
	24:0	[17] 2	—	—
**Beverages**				
Alcohol (*n* = 4)	20:0	—	[17] 2, [18] 5 , [19] 3	[30] 3
	22:0	—	[17] 2, [18] 5, [19] 3	[30] 3
	24:0	—	—	[17] 2[18] 5, [19] 3 , [30] 3
Coffee (*n* = 2)	20:0	[17] 2	—	[30] 3
	22:0	[17] 2	—	[30] 3
	24:0	[17] 5	—	[30] 3
Fruit juice (*n* = 1)	20:0	—	—	[30] 3
	22:0	—	—	[30] 3
	24:0	—	—	[30] 3
Sugar sweetened beverages (*n* = 1)	20:0	—	—	[30] 3
	22:0	—	—	[30] 3
	24:0	—	—	[30] 3

1 The numbers in the columns refer to the reference numbers in the main document.

2 Fatty acid was examined in both RBC and total plasma pool.

3 Fatty acid examined was within the phospholipid.

4 Fatty acid examined was within the RBC.

5 The fatty acid examined was within the total plasma pool.

6 Fatty acid examined within four different lipid pools: total plasma, non-esterified fatty acid, phospholipid, cholesteryl ester and triacylglycerol.

7 This study described saturated fatty acid/ polyunsaturated fatty acid intake from specific food groups including dairy, meat, fish, fish supplements, spreads and cooking fat.

8 Meat intake was estimated as habitual consumption of animal-based meat products (i.e., high meat eater, moderate meat, ovo-lacto vegetarians, vegans.

### Nutrients and food components

The included studies in this review examined the associations of a wide range of nutrients and individual food components with circulating VLCSFAs. Fat was the most commonly studied macronutrient [17, 19, 23, 24, 31], where 2 cross-sectional studies showed positive associations between total fat intake and 22:0 and 24:0 [17, 19]. Furthermore, Lemaitre et al. [19] reported a significant trend of increasing saturated fat consumption across quartiles of 20:0 and 22:0 (saturated fat % energy [Q1 of 20:0: 9.9% compared with Q4: 10.3% ] and [Q1 of 22:0: 9.8% compared with Q4: 10.4%], both *P*-trend < 0.002). Similarly, in a case-cohort study [29], saturated fatty acid intake was positively correlated with 22:0 and 24:0 (*r* = 0.20, *P* ≤ 0.003) when estimated using a validated 12-item questionnaire [32].

In addition, 3 cross-sectional analyses examined the associations of different types of saturated fat intake with circulating VLCSFAs [17, 23, 24], including dietary palmitic acid (16:0), stearic acid (18:0), 20:0, 22:0, and 24:0. Ardisson Korat et al. [17] showed that intake of 16:0 and 18:0 was positively correlated with plasma and red blood cell 20:0, 22:0, and 24:0 (r = 0.02–0.19) however, Furtado et al. [24] reported no correlation between dietary 20:0 and 22:0 with circulating phospholipids, triacylglycerols, nonesterified fatty acids, or cholesteryl ester 20:0 and 22:0. Lastly, Takkunen et al. [23] examined saturated fatty acids intakes from specific food sources such as fish, dairy products, meat and spreads, and cooking fat. The results indicated that saturated fat from dairy and spreads or cooking fats were positively correlated with red blood cells 22:0 (*r* = 0.086–0.132, *P* < 0.01) [23].

As for polyunsaturated fat, Takkunen et al. [23] reported a negative correlation between polyunsaturated fat from fish and fish oil supplements with 20:0 (*r* = –0.07, p<0.05) and 22:0 (*r* = –0.182, *P* < 0.0001), and there were no correlations with polyunsaturated fats from meat products [23]. In contrast, 2 other cross-sectional analyses examining polyunsaturated fat intake as a percent of total energy intake per day reported a positive correlation of polyunsaturated fat with 22:0 and 24:0 (*P* < 0.05) [17, 19]. Lastly, Ardisson Korat et al. [17] examined a range of other fat sources, including dairy, animal, and vegetable fats, of which dairy and vegetable fats had a positive correlation with plasma or red blood cell VLCSFAs (*r* = 0.04–0.17).

Carbohydrate and protein intakes were also reported in the studies included in this review. Lemaitre et al. [19] documented a lower consumption of carbohydrates in participants in higher quartiles of all VLCSFAs (carbohydrate intake, % energy [Q1 of 20:0: 53.5% compared with Q4: 52.1%], [Q1 of 22:0: 53.8% compared with 52.0%], [Q1 of 24:0: 53.7% compared with 52.2%], all *P*-trend ≤ 0.002) [19], and lower protein consumption in subjects in higher quartiles of 22:0 (protein intake, % energy [Q1 of 22:0: 19.2% compared with 18.7%], all *P*-trend ≤ 0.002). However, a pooled analysis of subjects from NHS and Health Professional Follow-Up Study (HPFS) showed conflicting results between the 2 cohorts [17], such that results from HPFS suggested a negative association between carbohydrate intake with VLCSFAs (*r* = –0.02–[–0.10]), while results from NHS showed a weak positive association (*r* = 0.01–0.06). Lastly, 1 study examined vitamin E intake from food sources and found no significant associations with plasma VLCSFAs [27].

### Foods and whole food groups

In addition to examining macro and micronutrients, multiple cross-sectional analyses and 1 longitudinal analysis explored the association of foods and whole food groups with circulating VLCSFAs (Table 2).

Three studies examined the association of meat intake with circulating VLCSFAs [17, 25, 30], and conflicting findings were shown depending on the type of meat. A range of methods was used to estimate meat consumption, and meat products were categorized differently. For example, Li [25] estimated total meat intake by separating subjects by high meat (≥280 g meat/d [uncooked weight]), moderate meat (<280 g/d), low meat (ovo-lacto diet: did not consume meat more than 6 times per year, but consumed eggs and dairy products) and no meat (vegan diet: consumed no meat and consumed eggs and dairy products less than 6 times per year) intake. The results indicated that subjects with low meat or no meat intake had a significantly lower concentration (mg/100 mL) of 20:0 compared to subjects grouped into high and moderate meat intake (20:0 [no/low meat: 0.1–0.2 mg/100 mL compared with high meat/ moderate meat: 0.3–0.4], *P* < 0.001) (25). On the other hand, in a longitudinal analysis over 13 years, Zheng et al. [30] examined the association of a wide range of meat groups, including red meat, white meat, processed meat, and liver, with phospholipid VLCSFAs. The authors reported that higher white meat intake was associated with an 11.3% lower concentration of phospholipid 20:0 and a 9.21% lower concentration of 24:0 (*P* < 0.05). Conversely, a higher intake of fatty fish was associated with a higher concentration of 20:0, and white fish intake was associated with a higher concentration of 24:0 [30]. No significant associations were reported for red, processed, and unprocessed meat and liver intake with circulating VLCSFAs [30]. Similarly, Ardisson Korat et al. [17] examined the association of unprocessed meat with VLCSFAs and found weak correlations.

Three studies examined the associations of nuts or legumes intake with VLCSFAs [17, 19, 30]; however, each study provided different definitions for the nuts and legumes food category. For example, Zheng et al. [30] conducted 2 individual analyses for legumes, and nuts and seeds (including peanuts and peanut butter), whereas Ardisson Korat et al. [17] conducted 3 separate analyses for peanuts, peanut butter, and other nuts. Lastly, Lemaitre et al. [19] conducted a single analysis on peanut intake with VLCSFAs without specifying whether peanut butter was included. Despite the different food items included in the nuts or legumes categories, all of the studies showed a positive association between nuts and seed or peanut intake with 22:0 and 24:0. However, most of the analyses did not find significant associations between peanuts, peanut butter, and nut intake with circulating 20:0 [17, 19, 30].

Lastly, 4 studies examined the association of beverage intake with VLCSFAs, including coffee, fruit juice, sugar-sweetened beverages, or alcohol [[17], [18], [19], 30]. Zheng et al. [30] showed no association between coffee, fruit juice, sugar-sweetened beverages, and alcohol intake and changes in any VLCSFAs over 13 years. Meanwhile, Ardisson Korat et al. [17] showed a positive correlation between coffee intake with all VLCSFAs (*r* = 0.05–0.10). Three cross-sectional analyses showed that alcohol consumption had a significant negative association with 20:0 and 22:0 [[17], [18], [19]].

### Smoking

Five studies reported on the relationship between smoking status and VLCSFAs [18, 19, 26, 28, 29] (Table 3). Three articles reported no correlation between smoking and VLCSFAs [19, 28, 29]. However, Zhao et al. [18] documented fewer smokers in the higher quartiles of 20:0 and 22:0, whereas Santos et al. [26] showed that heavy-smoking males (>20 cigarettes/d) had higher 24:0 compared to nonsmokers.

**Table 3:** Summary of 6 studies describing the correlation of smoking and physical activity with very long-chain fatty acids (VLCSFA)^1^

Reference	Study design	N, Age	Country	Fatty acid	Serum fraction (methods)	Fatty acid unit^2^	Evaluated factors	Results	Statistical analyses/covariates
Ardisson Korat et al., 2019 [17]	Prospective cohort study (Only cross-sectional analyses reported)	2854 (F:1392, M:1462), Mean age: 60.4-65y	USA	20:0 22:0 24:0	Plasma and RBC (GC-FID)	%	Physical activity (metabolic equivalents per week)	Baseline cross-sectional analysis from healthy participants: • VLCSFA was positively correlated with physical activity (*r* = 0.04-0.12)^6^	Spearman correlation, adjusted for age, total energy intake, BMI
Fernández-Real J et al., 2005 [29]	Cross-sectional	116 (M:76, F:40), Mean age: 36-40y	Spain	20:0 22:0 24:0	Plasma (GC-FID)	%	Smoking status (defined as at least 1 cigarette/day in the previous 6 months)	• No difference in VLCSFA between smokers and non-smokers (20:0(non-smokers: 0.30% vs Smokers: 0.28%), 22:0 (non-smokers:0.85% VS smokers:0.80%), 24:0(non-smokers:1.06% VS 1.04%))	T-test, Pearson correlation
Gellert et al., 2017 [28]	Cross-sectional	446 (F:446), Mean age 48.5y	Germany	20:0 22:0 24:0	RBC (GC)	%	Smoking (yes/ no)	• No significant association with smoking status 20:0(non-smokers: 0.16% vs smokers:0.16%), 22:0(0.32% vs 0.36%), 24:0(0.85% vs0.83%)	T-test
Lemaitre et al., 2015 [19]	Prospective cohort study (Only cross-sectional analyses reported)	3179 (M:38.6%), Mean age 75y	USA	20:0 22:0 24:0	PL (GC-FID)	%	Smoking status (current smoker), Physical activity (kcal/week)	Baseline cross-sectional analysis from healthy participants: • No difference in concentration of VLCSFA between smoking status • Physical activity and 22:0 and 24:0 were positively associated [(kcal/week (Q1 of 22:0: 1341 vs Q4: 1611), (Q1 of 24:0: 1200 vs Q4: 1717)]^4^	Linear regression
Santos et al., 1984 [26]	Cross-sectional	219 (M:219), Ages 40-62y	Spain	20:0 24:0	Plasma (GC)	%	Smoking (non-smokers, <10 cigarettes/day, 10–20 cigarettes/day, >20 cigarettes/day)	• No difference in 20:0 by smoking status 20:0(non-smokers:0.52%, heavy smokers (>20 cigarettes/day):0.49%) • 24:0 was higher in heavy smokers (non-smokers: 0.23% vs smokers: 0.34%)^3^	T-test
Zhao et al., 2018 [18]	Cross-sectional	1729 (M:586, F: 1143), Age range: 35-59y	China	20:0 22:0 24:0	Plasma (GC-FID)	%	Smoking status (at least one cigarette/ day), physical activity (at least once a week, excluding walking and riding)	• The higher quartiles of 20:0 and 22:0 had fewer smokers [(Q1 20:0: 123 smokers vs Q4: 88)3 and (Q1 22:0: 131 subjects vs Q4: 91)]^5^ • Physical activity did not differ between quartiles of VLCSFA	χ2 test

1 GC-FID: Gas chromatography – flame ionization detector; PL: Phospholipid; RBC: red blood cell; USA: United States of America.

2 Fatty acid are presented as total percent fatty acid (%) or in concentration.

3 *p* < 0.05.

4 *p* < 0.01 .

5 *p* < 0.001.

6 Did not specify p-value.

### Physical activity

Three studies examined the association of physical activity with VLCSFAs [[17], [18], [19]] (Table 3). All of the studies estimated physical activity using self-reported questionnaires: Ardisson Korat et al. [17] measured physical activity using metabolic equivalents per week, Lemaitre et al. [19] as kilocalorie expenditure over a week (kcal/week), and Zhao et al. [18] as the frequency of physical activity per week. Zhao et al. [18] reported no difference in the frequency of physical activity between quartiles of VLCSFAs. Similarly, Lemaitre showed no difference in physical activity across quartiles of 20:0; however, there was a higher caloric expenditure for participants in higher quartiles of 22:0 and 24:0 (kcal/wk [Q1 of 22:0: 1341 compared with Q4: 1611], [Q1 of 24:0: 1200 compared with Q4: 1717], both *P*-trend ≤ 0.002). Lastly, Ardisson Korat et al. [17] showed conflicting correlations between results from the NHS and HPFS cohorts. The HPFS showed a negative correlation between physical activity and plasma 20:0 (*r* = –0.04), while the NHS cohort showed a positive correlation (*r* = 0.06). Nonetheless, a consistent positive association was found for both cohorts between physical activity and plasma 22:0 and 24:0 (*r* = 0.04–0.11).

### Quality of included studies

Supplementary Tables 2 and 3 show the risk of bias assessment using the NOS. Eight of the 11 articles were scored ≥6 on the NOS scale, suggesting a low risk of bias. Because of the lack of adjustments of confounders in most of the extracted analyses, a majority of the studies scored low for the comparability assessments; nonetheless, the included studies scored high in the assessment of the exposure and outcome variables. Moreover, the only prospective cohort study had a low risk of bias (Supplementary Table 3). Overall, despite the low risk of bias scored in the NOS, the selective reporting of results relevant to the lifestyle correlates of VLCSFA may introduce additional bias.

## Discussion

To our knowledge, this is the first systematic review that summarizes current literature examining the relationship between diet, physical activity, and smoking on circulating VLCSFAs. Although our literature search identified 12 observational studies, each lifestyle variable was only evaluated for its association with VLCSFAs in 1 to 2 studies. Despite the limited literature for each identified lifestyle variable, the review found evidence that higher intakes of nuts and seeds, as well as total fat and certain fat subcategories, were positively correlated with circulating 22:0 and 24:0. Meanwhile, alcohol consumption was associated with lower 20:0 and 22:0. Furthermore, positive associations between physical activity and 22:0 and 24:0 were observed, but conflicting evidence was shown for associations between smoking and circulating VLCSFA.

### Dietary intake

Of the 9 studies that examined the association of dietary intake with VLCSFAs, 7 studies used validated self-reported FFQs to assess dietary intake [17, 19, [23], [24], [25], 30, 31], whereas the other 2 studies used in-person interviews or 4-day food diary [18, 27]. Although most of the studies used validated food assessment tools, the FFQs used may not have been validated for the specific food components contributing to VLCSFAs. Additionally, the timeframes of the FFQ (typically capturing usual intake in the prior 6 mo to 1 y) and the circulating VLCSFAs (reflecting more recent intake) may not have overlapped. Therefore, the associations presented may be underestimated due to misclassification bias.

In addition, given the inconsistencies in the classification of whole food groups across studies, it was challenging to identify all dietary correlates of circulating VLCSFAs precisely. For example, Zheng et al. [30] included nuts and peanut butter in the “nuts and seeds” food group, whereas Ardisson Korat et al. [17] examined peanut and peanut butter as 2 separate categories. Lastly, Lemaitre et al. [19] examined the correlation of peanuts with VLCSFAs but did not specify whether peanut butter was also included. Despite the different food items used to classify the legume and nut food groups, all analyses reported consistent evidence that intake of peanut butter, peanuts, nuts, and seeds was positively associated with 22:0 and 24:0. Peanuts and macadamia nuts are food sources that contain high amounts of 20:0 and 22:0 [33]. Previous intervention studies are consistent with these results as dietary intervention studies evaluating peanuts or macadamia nuts demonstrated an increase in the absolute concentration of plasma 20:0, 22:0, or 24:0 after short to long-term dietary intervention [[14], [15], [16]].

Furthermore, 2 studies showed a consistent positive correlation between total fat or saturated fat intake with circulating 22:0 and 24:0. This observation is consistent with a 6-wk-long randomly assigned controlled intervention reporting that participants in a moderate-fat intervention arm (34% energy from fat) had a higher concentration of 22:0 and 24:0 in both red blood cells and phospholipid compared to participants in the low-fat arm (17% energy from fat). Again, similar to our findings, there were no significant changes in 20:0 [34]. Although no mechanistic evidence explains the lack of changes in 20:0 from dietary intake, the current literature suggests that 22:0 and 24:0 may be more easily influenced by dietary fat intake.

Overall, although a range of foods and whole food groups were examined, the literature was generally limited by the heterogeneity of food group classification and often only 1 or 2 studies examined a specific dietary variable (Table 2). Given these limitations, it is difficult to draw firm conclusions on the impact of each nutrient or dietary variable on circulating VLCSFAs.

### Smoking and physical activity

Smoking is well known to be associated with altered lipid metabolism and increased serum concentrations of long-chain saturated fatty acids [35, 36]. Nevertheless, most of the studies included in this review showed no association between smoking status and VLCSFAs. One possible explanation for the lack of association might be the low number of subjects who were smokers. In the Cardiovascular Health Study (*n =* 3179), only 9% of participants were smokers [19]. In addition, most of the studies did not examine the dose-response effects of smoking on VLCSFAs, except for Santos et al. [26], who reported a significant difference in 24:0 between heavy smokers (>20 cigarettes/day) compared with nonsmokers. These results suggested that circulating VLCSFAs may only be affected by high exposure to cigarette smoking.

Conflicting results were reported regarding the association of physical activity with VLCSFAs. Two studies conducted in the United States used validated physical activity questionnaires [[37], [38], [39]] to estimate the intensity of physical activity using metabolic equivalents per week and kcal/wk [17, 19]; they showed a positive association between physical activity with 22:0 and 24:0 in the plasma and phospholipid pools [17, 19]. Meanwhile, a study conducted in China used an interview to collect self-reported frequency of physical activity per week [18] and found no significant associations. The conflicting results may be due to the different methods used to assess habitual physical activity (i.e., the inclusion of metabolic intensity in the studies conducted in the United States).

Overall, there is currently no mechanistic link between overall healthier lifestyle habits such as increased physical activity and lower alcohol consumption with circulating VLCSFAs; however, recent reviews suggest that higher VLCSFAs may be a biomarker of lower *de novo* lipogenesis and/or overall health status [40, 41]. Therefore, the positive association between healthier lifestyle habits and VLCSFAs might reflect the overall benefits of a healthy lifestyle and improved cardiometabolic health. On the other hand, results from the animal model and cell culture studies have led to the hypothesis that the unique biochemical characteristics of VLCSFAs may influence cell membrane integrity and insulin signaling, thereby playing a significant role in cardiometabolic health [42, 43]; nonetheless, the mechanisms underlying the association between VLCSFAs and cardiometabolic disease is not well understood. Thus, future well-designed randomly assigned control trials or mechanistic studies on modifiable lifestyle habits and VLCSFA metabolism are necessary to identify the determinants of VLCSFAs.

### Limitations of current studies

First, although a number of studies using a range of observational designs were included in this review, relevant data on VLCSFAs were mainly derived from cross-sectional analyses, with only 1 study examining the longitudinal association of dietary intake with changes in VLCSFAs [30]. Therefore, the temporal influence of dietary or lifestyle habits on circulating VLCSFAs could not be established. Future well-designed prospective cohort studies, randomly assigned controlled trials, and mechanistic investigations using isocaloric diets should be implemented to better understand the causal impact of specific dietary patterns, foods, or food components on circulating VLCSFAs. Second, most of the relevant data for this review were extracted from baseline bi-variate descriptive analysis, such as Pearson/Spearman correlations or comparisons of fatty acid distribution across categories. For example, all the studies examining associations between smoking and VLCSFAs were extracted from student t-test comparisons of fatty acid distributions between smokers and nonsmokers. In addition, the results from 8 of the 12 studies included in this review were not adjusted for potential confounding variables. Given that age, sex, total energy intake, and adiposity are potential variables that may confound the association between lifestyle variables with VLCSFAs, the results reported in this review are at risk of residual confounding. Third, the fatty acids were measured in different lipid compartments, namely, red blood cells, total plasma, phospholipid, triacylglycerol, nonesterified fatty acids, and cholesteryl ester pools. Fatty acids in separate lipid compartments may reflect different metabolic processes and dietary intake [24]; hence the results from different plasma compartments may not be directly comparable. Lastly, given the considerable heterogeneity of dietary variables examined and the various types of statistical tests used between studies, we could not conduct a meta-analysis to summarize the associations.

In conclusion, the findings of this systematic review indicate that dietary intake may have a moderate influence on circulating VLCSFAs. Specifically, peanuts, total fat, and certain sub-categories of fat may have a positive association with circulating 22:0 and 24:0; conversely, alcohol consumption has an inverse association with 20:0 and 22:0. There was also some evidence that higher physical activity may be associated with higher VLCSFAs; however, findings regarding the association of smoking with VLCSFAs were inconsistent. This systematic review highlights the lack of higher-quality observational studies examining the longitudinal association of lifestyle variables with circulating VLCSFAs. Given the emerging studies suggesting that VLCSFAs may be a biomarker of better health status, the literature may also benefit from studies examining the effects of adherence to beneficial dietary patterns (e.g., Mediterranean, Dietary Approaches to Stop Hypertension) and combined healthy lifestyle behaviors on these specific fatty acids [44]. In addition, prospective cohorts, randomly assigned control trials, and basic science research are warranted to better characterize the underlying pathways regulating circulating concentrations of VLCSFAs.

## Funding

KZHL was funded by an Ontario Graduate Scholarship. This project was funded by the Canadian Insitutes of Health Research.

## Author disclosures

AJH holds research funds from Dairy Farmers of Canada. All other authors report no conflicts of interest.

## Data availability

The papers included in the systematic review are from publicly available database.
