# Supplementary material for: Lifestyle Factors Associated with Circulating Very Long-Chain Saturated Fatty Acids in Humans: A Systematic Review of Observational Studies
Source: Adv Nutr. 2022 Dec 17;14(1):99–114. doi: 10.1016/j.advnut.2022.10.004 (PMC10102996; doi:10.1016/j.advnut.2022.10.004)
Supplement: Multimedia component 1 [file mmc1.docx]

Online supplementary material

**Supplementary Table 1. Search strategy**

|  | Medline |  | Embase |  | Cochrane Central Register of Controlled Trials |
| --- | --- | --- | --- | --- | --- |
| 1 | physical activit*.mp. | 1 | physical activit*.mp. | 1 | physical activit*.mp. |
| 2 | exp physical activity/ | 2 | exp physical activity/ | 2 | exp Motor Activity/ |
| 3 | exercis*.mp. | 3 | exercis*.mp. | 3 | exercis*.mp. |
| 4 | exp exercise/ | 4 | exp exercise/ | 4 | exp exercise/ |
| 5 | smoking*.mp. | 5 | smoking*.mp. | 5 | smoking*.mp. |
| 6 | exp smoking/ | 6 | exp smoking/ | 6 | exp smoking Cessation/ |
| 7 | lifestyle*.mp. | 7 | lifestyle*.mp. | 7 | lifestyle*.mp. |
| 8 | exp lifestyle/ | 8 | exp lifestyle/ | 8 | exp lifestyle/ |
| 9 | diet therap*.mp. | 9 | diet therap*.mp. | 9 | diet therap*.mp. |
| 10 | exp Diet Therapy/ | 10 | exp Diet Therapy/ | 10 | exp Diet Therapy/ |
| 11 | diet modification*.mp. | 11 | diet modification*.mp. | 11 | diet modification*.mp. |
| 12 | exp Nutrition Assessment/ | 12 | exp Nutrition Assessment/ | 12 | exp Nutrition Assessment/ |
| 13 | exp Nutrition Therapy/ | 13 | exp Nutrition Therapy/ | 13 | exp Nutrition Therapy/ |
| 14 | Nutrition.mp. | 14 | Nutrition.mp. | 14 | Nutrition.mp. |
| 15 | alcohol consumption.mp. | 15 | alcohol consumption.mp. | 15 | alcohol consumption.mp. |
| 16 | exp Alcohol Drinking/ | 16 | exp Alcohol Drinking/ | 16 | exp Alcohol Drinking/ |
| 17 | healthy*.mp. | 17 | healthy*.mp. | 17 | healthy*.mp. |
| 18 | exp Health Behavior/ | 18 | exp Health Behavior/ | 18 | exp Health Promotion/ |
| 19 | **or/1-18** | **19** | **or/1-18** | **19** | **or/1-18** |
| 20 | very-long-chain fatty acid.mp. | 20 | very-long-chain fatty acid.mp. | 20 | very-long-chain fatty acid.mp. |
| 21 | VLCFA.mp. | 21 | VLCFA.mp. | 21 | VLCFA.mp. |
| 22 | arachidic acid.mp. | 22 | arachidic acid.mp. | 22 | arachidic acid.mp. |
| 23 | behenic acid.mp. | 23 | behenic acid.mp. | 23 | behenic acid.mp. |
| 24 | lignoceric acid.mp. | 24 | lignoceric acid.mp. | 24 | lignoceric acid.mp. |
| 25 | VLSFA.mp. | 25 | VLSFA.mp. | 25 | exp Eicosanoic Acids/ |
| 26 | VLCSFA.mp. | 26 | VLCSFA.mp. | 26 | tetracosanoic acid.mp. |
| 27 | exp Eicosanoic Acids/ | 27 | exp Eicosanoic Acids/ | **27** | **or/20-26** |
| 28 | tetracosanoic acid.mp. | 28 | tetracosanoic acid.mp. | **28** | **19 and 27** |
| 29 | **or/20-28** | **29** | **or/20-28** |  |  |
| 30 | exp cohort studies/ | 30 | exp cohort analysis/ |  |  |
| 31 | cohort$.tw. | 31 | exp longitudinal study/ |  |  |
| 32 | controlled clinical trial.pt. | 32 | exp prospective study/ |  |  |
| 33 | epidemiologic methods/ | 33 | exp follow up/ |  |  |
| 34 | limit 34 to yr=1966-1989 | 34 | cohort$.tw. |  |  |
| 35 | exp case-control studies/ | 35 | exp case control study/ |  |  |
| 36 | (case$ and control$).tw. | 36 | (case$ and control$).tw. |  |  |
| 37 | (case$ and series).tw. | 37 | exp case study/ |  |  |
| 38 | case reports.pt. | 38 | (case$ and series).tw. |  |  |
| 39 | (case$ adj2 report$).tw. | 39 | case report/ |  |  |
| 40 | (case$ adj2 stud$).tw. | 40 | (case$ adj2 report$).tw. |  |  |
| 41 | **or/31-33,35-41** | 41 | (case$ adj2 stud$).tw. |  |  |
| 42 | **19 and 29 and 41** | **42** | **or/30-41** |  |  |
|  |  | **43** | **19 and 29 and 42** |  |  |
|  |  |  |  |  |  |

For all databases, the original search date was in March 2021; an updated search was performed in February, 2022. The search was limited to human observational cohort studies and had no language restrictions.

**Supplementary Table 2. NOS study quality assessment for 11 cross-sectional studies**

| Study | Selection (max 5) | | | | Comparability (max 2) | Outcome (max 3) | | Total score^†^ |
| --- | --- | --- | --- | --- | --- | --- | --- | --- |
|  | Representativeness of the sample | Sample size | Non-respondents | Ascertainment of the exposure | Confounding factors controlled* | Assessment of outcome | Statistical test |  |
| Ardisson Korat et al. (7) | 0 | 1 | 1 | 2 | 2 | 2 | 1 | 9 |
| Fernández-Real et al. (27) | 0 | 1 | 0 | 2 | 0 | 2 | 1 | 6 |
| Furtado et al. (22) | 0 | 1 | 0 | 2 | 2 | 2 | 1 | 8 |
| Gellert et al. (26) | 0 | 1 | 0 | 2 | 0 | 2 | 1 | 6 |
| Lemaitre et al. (29) | 1 | 1 | 0 | 1 | 0 | 2 | 1 | 6 |
| Lemaitre et al. (17) | 1 | 1 | 0 | 2 | 0 | 2 | 1 | 7 |
| Li. (23) | 0 | 1 | 0 | 1 | 0 | 2 | 1 | 5 |
| Santos et al. (24) | 0 | 1 | 0 | 2 | 0 | 2 | 1 | 6 |
| Takkunen et al. (21) | 1 | 0 | 0 | 1 | 0 | 2 | 1 | 5 |
| Zhao et al. (25) | 1 | 1 | 0 | 1 | 2 | 2 | 1 | 8 |
| Zhao et al. (10) | 0 | 1 | 0 | 1 | 0 | 2 | 1 | 5 |

*For one-point, confounding variables including age and sex must be assessed. For two points, additional confounding variables on BMI/ body weight and total caloric intake must be assessed.

† A maximum of 10 points may be awarded, with a score of 6 or more being considered higher quality.

**Supplementary Table 3. NOS study quality assessment for 1 prospective study**

|  | **Selection (max 4)** | | | | **Comparability (max 2)** | | **Outcome (max 3)** | | | Total score† |
| --- | --- | --- | --- | --- | --- | --- | --- | --- | --- | --- |
| Study | Representativeness of exposed cohort | Selection of non-exposed cohort | Ascertainment of exposure | Outcome of interest was not present at start of study | Adjust for the most important risk factors | Adjust for other risk factors | Assessment of outcome | Follow-up length | Loss to follow-up rate |  |
| **Zheng et al. (28)** | 1 | Not applicable* | 1 | Not applicable* | 1 | 1 | 1 | 1 | 1 | 7 |

*This criterion is not applicable to the research question because the outcome of the study is circulating fatty acids and there are no “non-exposed” cohort.

*This criterion is not applicable because of the outcome of interest in this study is circulating fatty acid.

†A maximum of 9 points may be awarded, with a score of 6 or more being considered higher quality.
